# Effects of Early Resistance Training After Liver Transplantation Procedures: A Randomized Controlled Pilot Trial

**DOI:** 10.5152/tjg.2022.21959

**Published:** 2022-10-01

**Authors:** Tuba Yüksel Ergene, Didem Karadibak, Ramazan Dönmez, Kâmil Yalçın Polat

**Affiliations:** 1 Department of Cardiopulmonary Physiotherapy and Rehabilitation, Memorial Ataşehir Hospital, İstanbul, Turkey; 2 Department of Cardiopulmonary Physiotherapy and Rehabilitation, Dokuz Eylül University, Faculty of Physical Therapy and Rehabilitation, İzmir, Turkey; 3 Department of General Surgery, Medicana Ataşehir Hospital, İstanbul, Turkey; 4 Department of General Surgery, Head of Organ Transplantation Center, Memorial Ataşehir Hospital, İstanbul, Turkey

**Keywords:** Exercise capacity, fatigue, liver transplantation, muscle strength, resistance training

## Abstract

**Background:**

Exercise interventions improve muscle performance and functionality when applied more than 6 months after liver transplantation, but no studies have reported on earlier exercise interventions. Hence, we assessed the effects of early resistance training on functional outcomes in adult liver recipients.

**Methods:**

The study included 30 liver transplantation patients (53.2 ± 12.4 years) randomly assigned to a training group (n = 15) or a control group (n = 15). Data collected preoperatively and 4 and 8 weeks post-surgery were analyzed, including peripheral and respiratory muscle strength, exercise capacity, physical performance, and fatigue. An 8-week physiotherapy program was applied (training group: standard physiotherapy + resistance training; control group: standard physiotherapy) for 2 sessions/day, 5 days/week.

**Results:**

Baseline data showed a homogeneous distribution in the between-group comparisons. In the within-group analysis; EG showed higher improvements in physical performance (TG: *P* = .001, CG: *P* = .048) and fatigue perception (TG: *P* = .001; CG: *P* = .006), than the CG. The TG showed eight-week improvements in exercise capacity, peripheral muscle strength, and maximal inspiratory pressure (*P* = .001), and maximal expiratory pressure (*P* = .047), while CG remained unchanged (*P* > .05). In the between-group analysis; the improvements indicated significant differences in deltoid strength and fatigue perception, in favor of the TG (*P* < .05). A change of 0.9 kg in peripheral muscle strength and >37.8 m in 6-min walk distance (6MWD) was determined, representing clinically significant improvement in liver recipients.

**Conclusion:**

Early resistance training may improve muscle strength, exercise capacity, physical performance, and fatigue perception in liver recipients, when added to standard physiotherapy. The estimated minimal clinically important differences are meaningful to clinicians in setting liver transplanted patient-specific goals.

**Main Points**

- To the best of our knowledge, this is the first study demonstrating that a resistance-training program implemented in the *early* phase of liver transplantation (LT) can have clinically relevant effects on exercise capacity, peripheral and respiratory muscle strength.
- The first evidence that integrating resistance training into standard physiotherapy approaches for liver recipients also contributes to the improvement of physical performance and decline in fatigue perception in the early posttransplant period.
- The estimated minimal clinically important difference values of the functional parameters will be helpful in setting individualized goals for liver recipients.

## Introduction

Liver transplantation (LT) is a life-saving procedure performed as a major intra-abdominal intervention for patients with end-stage liver disease (ESLD). There has been a dramatic increase in the number of transplants because of the many causes of liver disease, with approximately 28 000 liver transplants performed worldwide in 2015.^1^ In Turkey, there were 13 432 ESLD cases that involved liver transplants in the 10 years from the start of 2012 to the end of October 2021(accessed 29 October 2021).^2,3^ Although survival rates and quality of life among liver recipients have improved, LT still has a high risk of complications, including preoperative losses of muscle mass and physical deconditioning, post-surgical complications (intra-abdominal/incisional), fatigue, and poor exercise capacity, all of which negatively affect functional recovery.^4,5^ The use of immunosuppressant medication also contributes to the development of age-related declines in muscular strength and physical ability,^6^ which have been consistently associated with posttransplant functional deficits. The quality and quantity of skeletal muscle mass have also been closely related to posttransplant mortality in liver recipients.^7^

The overall goal of posttransplant rehabilitation programs for solid-organ recipients is to help them regain function by increasing general mobility in the early posttransplant period and improve functional exercise capacity, muscle strength, and health-related outcomes in their daily lives.^8^ Various exercise-based interventions involving aerobic and resistance training or physical activity counseling have been shown to improve physical performance in tasks, muscle strength, and the physical domain of life in adults who have received liver transplants.^9,10^ However, these studies have focused on the >6-month postoperative period; furthermore, studies that examined exercise training effects in the early post-surgical period involved different patient populations, such as colorectal resection and hip replacement patients.^11,12^ Therefore, we undertook a prospective randomized controlled pilot trial to investigate the effects of an *early* progressive resistance training program on exercise capacity, muscle strength, and fatigue in adult liver recipients and compare them to the effects of standard physiotherapy care.

## Materials and Methods

### Study Design and Participants

This pilot study used a prospective randomized controlled design and included 30 patients who had undergone LT at the Organ Transplantation Centre of Memorial Ataşehir Hospital, Turkey, between September 2018 and June 2019. Ethical approval was granted by the Non-Interventional Research Ethics Committee of Dokuz Eylül University (approval number: 2019/18-27). The trial protocol and statistical analysis plan were registered before conducting the research and accepted for publication before unblinding by the Health Sciences Institutional Registry System. The study adhered to the Declaration of Helsinki. Eligible patients who accepted to participate in the study voluntarily signed the informed consent form.

The participants were randomly assigned to either a training group (TG) (standard physiotherapy + resistance training) or a control group (CG) (standard physiotherapy). A randomized design was used with a 1 : 1 ratio. Randomization was performed by an independent researcher, who was not otherwise involved in the study, via an automated, web-based random integer generator (random.org) based on true randomness. Participants, family members, and ward staff were not informed of group allocation; analysis was performed by a blinded statistician.

Patients were eligible for inclusion if they were 18 years of age or older, had completed all the preoperative physiotherapeutic evaluation procedures, were hemodynamically stable and spontaneously breathing postoperatively, and could read, write, and understand Turkish. Patients were excluded if they had comorbid conditions that would affect their exercise performance (e.g., a lung pathology requiring regular use of a bronchodilator or neuro-musculoskeletal complications/limitations requiring an assistive device), difficulty following verbal instructions, a history of multi-organ transplantation, or if they were undergoing re-transplantation.

### Sample Size

The sample size calculation was based on data obtained from the study, using a combined intervention of exercise and dietary counseling after LT, and outcomes of showing greater increases in VO_2peak_ (*P *= .036) and self‐reported general health (*P *= .038) than for patients receiving the usual care.^13^ The sample size was calculated using the G*Power 3.1.9.6 software, with a 95% CI and 80% power. It was estimated that the study required 36 subjects (18 per group).

### Data Collection

The same researcher performed all physiotherapy evaluations and exercise interventions. Outcomes were measured at baseline (preoperatively) and 4 and 8 weeks after LT. All evaluations took about 40 minutes. Sociodemographic and clinical characteristics were collected in face-to-face interviews. Model for End-Stage Liver Disease (MELD) scores were calculated according to the existing system used by the hospital’s clinical laboratory. The transplant team evaluated each subject’s medical and surgical factors for candidacy. The American Society of Anesthesiologists class data (a summary measure of the physical status of the patient) evaluated by the anesthesiologist preoperatively was obtained from the medical records.

The 6-minute walk test was used to determine exercise capacity.^14^ The distance covered in 6 minutes was recorded as the 6-minute walk distance (6MWD) in meters. The predicted (pred) 6MWD was calculated for each participant using reference equations.^14^ Heart rate and oxygen saturation were measured before, during, and after each test. Dyspnea and perceived leg fatigue levels were determined using the Modified Borg Scale.^15^

Peripheral muscle strength, comprising knee extension (quadriceps) and shoulder flexion (anterior deltoid) and abduction (middle deltoid) measurements, was assessed using a hand-held dynamometer (Power Track Commander II). Three sustained maximal isometric contractions were performed in the previously described testing positions.^16^ A rest period of 30 seconds was given between each trial. The highest value during the preserved maximum tension over 1 second was recorded (kg), and pred values were interpreted.^16^

Respiratory muscle strength, comprising maximal inspiratory pressure (MIP) and maximal expiratory pressure (MEP) measurements, was assessed using a portable electronic mouth pressure device (Micro Mouth Pressure Measurement). Test maneuvers were performed 3 times; the highest value was recorded as cmH_2_O, which is also expressed as a percentage of pred values.^17^

The 30-second sit-to-stand test (STST) was used to measure physical performance and included sitting, mobility, rising, sit-to-stand, and standing components. Each test was performed twice, in a standardized order, with 5-minute rest periods. The number of times a participant stood in 30 seconds was assessed (with arms folded across the chest); the highest score was recorded. Higher STST scores were associated with higher performances based on the following equations for participants <50 and ≥50 years of age.^18^

Fatigue perception was assessed using the validated Turkish version of the Checklist Individual Strength (CIS-T).^19^ This 20-item questionnaire is a self-reported multidimensional instrument used to assess fatigue severity (subjective), concentration problems, reduced motivation, and activity level. Each item is scored on a 7-point Likert-type scale. Higher scores indicate a higher level of fatigue, ranging from 0 to 100; the 35-point cut-off score for severe fatigue was used.^19^

### Procedure

The TG received 8 weeks of resistance training that targeted deltoid and quadriceps as major limb muscles. Because the training was focusing on specific functional muscle groups, handgrip strength, which is used as a global measure of strength, was not included in the assessment. The authors developed the training protocol (2-3 sets of 6-10 repetitions [rep], with 1-2 minute rest between sets), and two 20-min sessions/day were conducted 5 days/week.

Resistance was generated by using a series of 150 cm-long elastic bands that provided increasing intensity. Intensity was gradually increased based on individual ability. Exercise load was established at a light-to-moderate intensity using the Borg Scale.^11^ Exercises were applied in the chair-sitting position (recommended) or sitting on the edge of the bed. The training program also comprised functional exercises that started with a half squat (5 reps daily) and progressed to sit-to-stand chair exercises (5 reps twice a day) according to the physical fitness level. The training program was carried out under supervised conditions during the first 2 weeks (hospital stay): vital signs were monitored and subjective fatigue levels and exercise-related pain were documented before, during, and after each session. All patients were trained before discharge on how to perform their training regimen safely without supervision at home for the remaining 6 weeks, and they received a patient-specific schedule to continue their training program. Patients were phoned weekly to ensure adherence and that there were no adverse effects.

Both groups received the following standard supervised physiotherapy follow-up, which is part of the posttransplant care at our center: preoperative education and postoperative respiratory physiotherapy, active/active assistive exercises, and early mobilization. Respiratory physiotherapy included positioning, lung expansion maneuvers, bronchial hygiene techniques, and incentive spirometer use. Graded early mobilization was initiated when participants were clinically stable.^20^ Participants were advised on coping with daily tasks and educated on a home-based discharge program, considering individual rehabilitation needs and graded activity principles. Follow-up calls were provided weekly to maintain the 8-week walking and to schedule appointments.

Standard general anesthesia and surgery protocols were used for all patients. The incision of choice was the Mercedes type, and the modified piggyback method was used. Antibiotic prophylaxis was routine, and all were on tacrolimus. Pain relief was provided according to the analgesic requirement. Blood transfusion was considered at a hemoglobin concentration of ≤8 g/dL and with international-normalized ratio monitoring. Posttransplant outpatient follow-ups were conducted.

### Statistical Analysis

Categorical variables are presented as percentages. Continuous variables are shown as mean and standard deviations (SDs) when normal distribution is assumed. When not assumed, median and quartiles are shown. The Shapiro–Wilk test was used to assess normal distribution in each continuous variable. The demographic and clinical characteristics of both groups were compared with chi-square in categorical variables with Yates continuity correction or Fisher’s exact test when needed. A *t*-test or Mann–Whitney *U* test was used to assess the difference in preoperative measurements between the TG and CG. The differences between preoperative and postoperative 4- and 8-week measurements were tested with the Wilcoxon Signed Rank Test for each pair, and respective effect sizes were estimated. Statistical Package for the Social Sciences 22.0 (IBM Corp.; Armonk, NY, USA) was used for statistical tests. Alpha error levels lower than 0.05 were accepted as significant. The effect sizes were estimated using an online tool where the data could be uploaded.^21^ Minimal clinically important difference (MCID) is the SD of the baseline measure (preoperative); by convention, a 20% change in the SD was defined as a small effect, 50% as a moderate effect, and 80% as a large effect using the distribution-based methods.^22^

## Results

### Demographic and Clinical Characteristics

**Figure 1.**
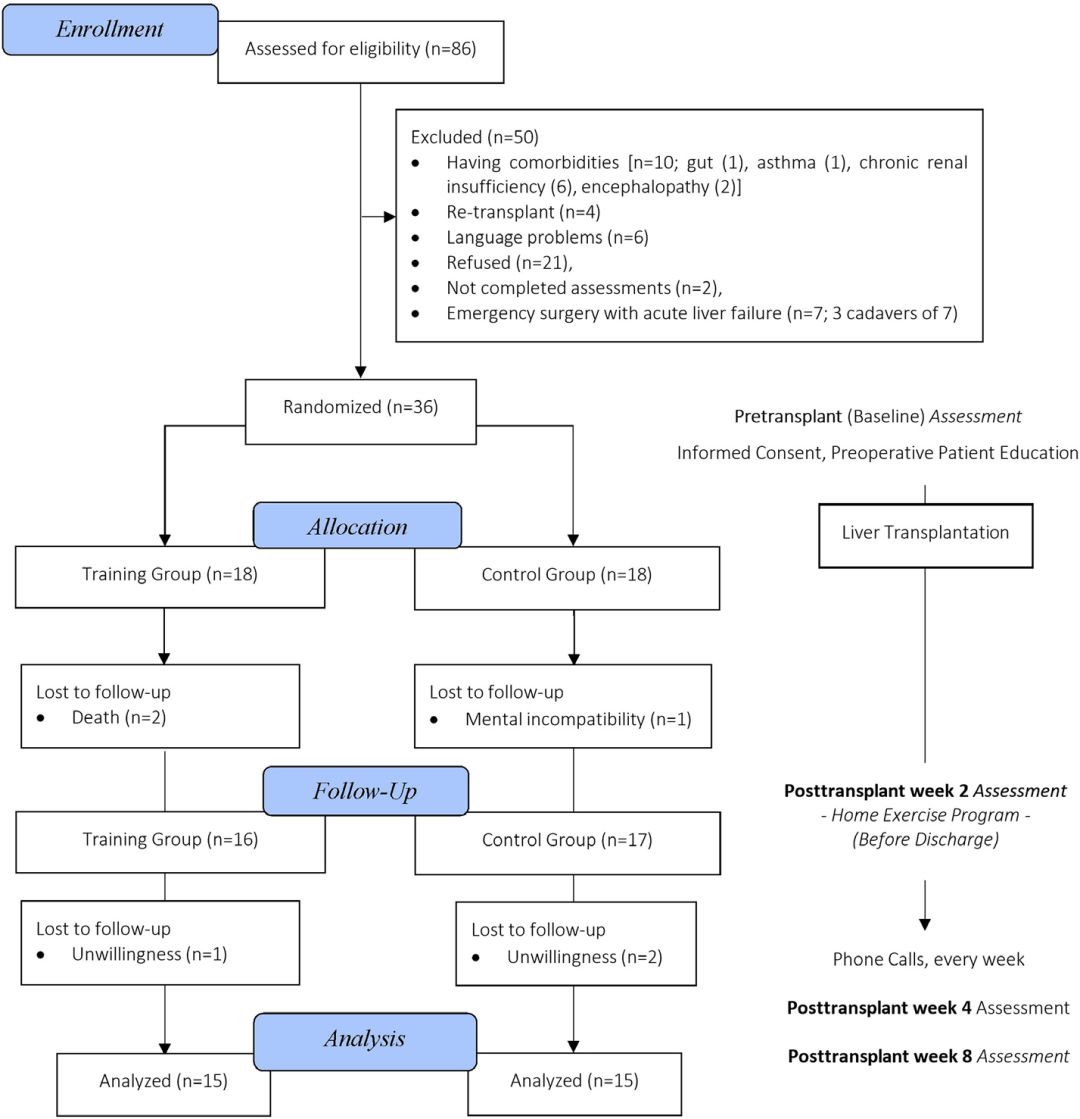
Study flow diagram.

A total of 86 patients scheduled to undergo LT were considered to participate (Figure 1). Of these patients, 36 were randomly assigned to either the TG or CG. After their surgery, 6 patients withdrew from the study (3 voluntarily withdrew, 1 was unwilling to participate, and 2 died). The patients who died were assigned to the TG. The causes of death were hepatic artery thrombosis and sepsis, which were diagnosed in the presence of multiorgan failure in the first posttransplant week as a surgical complication. They did not experience any adverse acute effects during or after the exercise sessions. Therefore, 30 liver recipients (53.2 ± 12.4 years) were enrolled for the final analysis.

Participants were tested preoperatively at an average of 2.1 ± 3.2 days before surgery. Their exercise behavior was assessed, and they were deemed physically active if they were active at least 3 days per week for 40 minutes or more. There were no statistical differences between the groups’ demographic and clinical characteristics (*P *> .05; Table 1). The mean waiting time for LT was 37.6 ± 51.46 months. The most common etiological causes of liver failure in the TG were cryptogenic cirrhosis (20%), alcoholic cirrhosis (13.3%), and viral hepatitis, hepatocellular carcinoma, or both (53.33%); the most common causes in the CG were cryptogenic cirrhosis (40%) and alcoholic cirrhosis (33.3%) (*P *= .375). All recipients included in the study had living liver donors as a result of providing time to perform their preoperative assessments.

The recipients were immediately extubated when hemodynamically stable within 15.3 ± 6.1 hours (range: 11-44 hours). The most frequent complication was pleural effusion, occurring in the first posttransplant days, which was not related to the resistance training. The postoperative 30-day mortality was 6.6%, with sepsis as the main leading cause. Patient compliance was high, and there were no adverse events associated with the resistance-training program. Physiotherapy sessions had 96.8% adherence (14/450 were not completed) during hospitalization; non-adherence was mainly associated with abdominal drain leaks and loss of vitality. Corridor walks were initiated mostly on postoperative day 2 (83.3% of patients) and progressed from 60 m with a daily increase based on patient ability. There were no statistically significant differences between groups in intensive care length of stay (TG: 1.9 ± 0.9 days, CG: 1.7 ± 1.0 days; *P *= .700), hospital stay (TG: 19.8 ± 6.9 days, CG: 19.3 ± 10.3 days; *P *= .868), and 30-day readmission (TG: 3.1 ± 4.1 days, CG: 4.7 ± 4.2 days; *P *= .299).

### Resistance Training Effects on Clinical Outcomes

In the TG, the 6MWD had increased from the preoperative baseline by 60.33 m at week 4 and 109.87 m at week 8 (reaching 72.9%, *P *= .017 and 82.4%, *P *= .001, of pred, respectively). Although the 6MWD improved in the CG, the difference was not statistically significant. Also, when the groups were compared, there was no significant difference in the 6MWD (*P *> .05, Table 2). An improvement of >37.8 m was determined to be the first evidence of MCID in 6MWD among liver recipients (Table 3). According to the obtained data, 86.7% of cases in the TG (13 patients) achieved MCID in 6MWD, representing a moderate clinically important improvement (with an effect size of 0.5).

The TG had improved major limb muscle strengths at week 8 posttransplant compared to the baseline and week 4 values (*P *= .001). The TG also showed increased MIP at week 8 posttransplant compared to the baseline and week 4 values (*P *= .016 and *P *= .001, respectively). An important difference was also found in MEP values in the TG at week 8, while CG remained unchanged (*P *= .047). However, in the CG, the major limb and respiratory muscle strengths recorded during the 8-week period were not significantly different from the baseline (*P *> .05). Between-group significant differences were found in shoulder flexion and shoulder abduction in favor of the TG after the 8-week training period (*P *= .019 and *P *= .021, respectively) (Table 2). The estimated MCID was 0.9 kg for shoulder flexion, shoulder abduction, and knee extension. Distribution-based meaningful differences are shown in Table 3. In the TG, 93.3% of the participants (14 patients) reached the MCID in muscle strength (effect size = 0.5).

Regarding the STST, in the TG, the score was higher at week 4 and week 8 posttransplant than at baseline. However, in the CG, an important difference was observed in the STST score at week 8 compared to the baseline and week 4 values (*P *< .05). When the TG and CG STST scores were compared, there was no statistically significant difference (*P *= .082).

The CIS-T score was lower in the TG than in the CG at week 8 (*P *< .05). The difference between the 2 groups was also significant and in favor of the TG (*P *= .009) (Table 2).

## Discussion

To the best of our knowledge, this is the first study demonstrating that a resistance-training program implemented in the *early* phase of LT can have clinically relevant effects on exercise capacity, peripheral and respiratory muscle strength, physical performance, and fatigue. The study showed that resistance training, in addition to standard physiotherapy, significantly improved functional outcomes of liver recipients. The between-group differences for deltoid strength and fatigue perception were statistically significant. Increases of 37.8 m in 6MWD and 0.9 kg in the major limb strength were determined to be the MCIDs for liver recipients.

Degenerative skeletal muscle loss is associated with increased mortality, morbidity, physical disability, and inferior quality of life before and after LT.^7^ Reversing muscle loss is challenging, and for this reason, the post-LT prognosis is generally poor.^23^ Resistance training is an effective countermeasure to muscle loss aimed at achieving progressive, positive adaptations.^24^ Van den Berg-Emons et al^9^ included liver recipients in a 12-week combined exercise program ≥1 year after transplant, which resulted in higher knee flexion strength. A 24-week post-LT exercise intervention using a similar training method improved hip extension, elbow flexion, overall maximal strength, and physical functioning.^10^ One cohort also reported 6-month posttransplant knee strength increasing from 60% to 100% through LT-combined supervised exercise.^25^ Although exercise interventions combining aerobic and resistance training are reasonable, there is a lack of early posttransplant evidence of their success.^10,26^ The present study shows that integrating resistance training into standard physiotherapy in the early postoperative period improves both peripheral and respiratory muscle strength. Also, the between-group improvements in deltoid muscle strength were statistically significant. In our opinion, patients were more tolerant of upper extremity exercises because of post-surgery abdominal drains and incisional pain. Additionally, an increase of at least 0.9 kg in muscle strength may indicate a clinically meaningful improvement among post-LT patients. Therefore, this study might serve as a helpful resource for future studies.

Concurrent resistance and aerobic exercises have been shown to improve aerobic capacity in LT patients.^9,10^ A similar exercise program applied in a liver transplant case resulted in increased skeletal muscle mass, walking capacity, and muscle strength, contributing to independent performance of daily living activities.^27^ However, these studies were late-term trials conducted at least 6 months post-LT. Beyer et al^25^ reported that supervised exercise led to improvements in physical fitness and functional performance 1 month post-LT. Given that early progress in exercise capacity enhances short-term rapid posttransplant recovery,^28^ this first report of resistance training plus standard physiotherapy improving functional exercise capacity after LT demonstrates the potential of this approach. The patient demographics, including MELD scores, were similar in this cohort. Although 6MWD plays a crucial role in the assessment of exercise capacity in LT subjects,^25^ it is unclear if the positive changes in muscle performance impact the functional level. This paper reveals that a >37.8 m change in 6MWD is clinically significant in liver recipients.

Transplant candidates experience accelerated functional decline, and changes in physical performance status should be considered predictors of functional capacity. Thus, this creates an opportunity to manage the posttransplant process through the trajectory of changes in physical function. Even though large randomized controlled trials are required, on the basis of existing reports, a combination of aerobic (3 days/week) and resistance exercises (2 days/week) at a moderate-high intensity is recommended to enhance physical performance while waiting for LT.^29^ The current findings show that training improves physical performance status. The significant, albeit much lower, improvements observed without training were concluded to be a result of the transplant, which enabled a rapid recovery of liver synthesis and metabolic functions.

In recent years, given the operative morbidity, surgical complications, and long‐term immunosuppression required, exercise training and physical activity counseling are promising approaches for reducing fatigue among liver recipients.^9^ Chascsa et al^30^ reported that the major patient-perceived limiting factor to exercise was fatigue, reported by 70%, followed by ascites and medications in LT candidates. While there is no easy solution (e.g., an effective medication) to overcoming fatigue for patients with liver diseases, exercise interventions, including structured training programs and being physically active, could positively influence fatigue perception by promoting muscle performance, activity participation, and daily functioning. In this study, while all participants experienced lower fatigue postoperatively, the TG’s fatigue perception improved by 28.1% at week 4 and 43.1% at week 8 post-surgery. Hence, the resistance training significantly improved fatigue perception. This may be due to a general lower fatigue perception among TG members; however, there was no statistically significant difference in preoperative fatigue perception between the groups. It is also worth mentioning that a transplantation patient population-specific tool was not used because no such questionnaire exists.

There are strengths and limitations in this study. In terms of strengths, this is the first study to prospectively confirm that early posttransplant resistance training positively affects exercise capacity, muscle strength, physical performance, and perceived fatigue among liver recipients. The estimated MCID values of the functional parameters will be helpful in setting individualized goals for liver recipients.

The 2 major limitations of this study are that part of the 8-week program was unsupervised and the small sample size. Another limitation is that this study included a very specific patient set; participants had relatively low MELD scores (<20) and received living donor liver transplants; thus, they tended to be physically better than those who had longer waits for cadaveric donors. Also, the reliability of the exercise program was not evaluated; however, the lack of significant adverse events experienced during the training program implies that it can improve functional outcomes under supervision and be easily applied at an affordable cost. This was a pilot study intended to guide further phase-II blinded research. Longer follow-up (e.g., 6-12 months) will allow analysis of the long-term effects of the intervention on cumulative outcomes, such as activities of daily life, functional level, and health-related quality of life.

In conclusion, this randomized controlled trial provides the first evidence that integrating resistance training into standard physiotherapy approaches for liver recipients provides therapeutic benefits and improves early posttransplant functional outcomes. The findings of this pilot study must be complemented by future studies that examine the long-term effects of integrating resistance training and that include larger cohorts.

## Figures and Tables

**Table 1.** Baseline Demographic and Clinical Characteristics of the Participants

**Variables**	**TG (n = 15)**	**CG (n = 15)**	*P*
Age, years (mean ± SD)	51.5 ± 15.4	54.8 ± 8.7	.482^b^
BMI, kg/m^2^ (mean ± SD)	26.0 ± 5.0	27.6 ± 3.8	.328^b^
Gender (F, %)	40	26.7	.439^a^
Educational level (≤secondary school, %)	53.3	40	.710^a^
Smoking habit (current user, %)	26.7	33.3	.755^a^
Alcohol use (current user, %)	20	33.3	.520^a^
Ascites (%)	60.0	46.7	.464^a^
Esophageal varices (%)	13.3	60	.008^a^
ASA status (%)			
III	66.7	53.3	.456^a^
IV	33.3	46.7
Exercise habit (%)			
Yes	40	33.3	.716^a^
Comorbidities (%)			
HT	13.3	26.7	.651†
DM	40	40	1.000^a^
Dyslipidemia	40	66.7	.143^a^
Coronary stent	*-*	13.3	.483†
Crohn	-	6.7	.50†
Operation duration, minutes (mean ± SD)	463.2 ± 53.4	498.6 ± 81.5	.172^b^
Intubation duration, minutes [M (IR)]	800.0 (200.0)	810.0 (235.0)	.787^c^
MELD score (mean ± SD)	18.9 ± 6.9	16.5 ± 5.0	.272^b^
Shoulder flexion, kg (mean ± SD)	11.2 ± 2.8	11.6 ± 3.8	.744^b^
Shoulder flexion pred (mean ± SD)	51.7 ± 9.9	48.3 ± 10.4	.375^b^
Shoulder abduction, kg [M (IR)]	11.0 (3.0)	10.0 (6.0)	.644**c**
Shoulder abduction pred [M (IR)]	50.0 (17.0)	51.0 (19.0)	.547^c^
Knee extension, kg (mean ± SD)	10.3 ± 2.2	11.7 ± 3.1	.168^b^
Knee extension pred (mean ± SD)	28.3 ± 6.9	29.1 ± 5.9	.698^b^
MIP, cmH_2_O (mean ± SD)	64.0 ± 31.9	69.4 ± 18.0	.573^b^
MIP pred (mean ± SD)	63.5 ± 28.4	65.6 ± 11.8	.790^b^
MEP, cmH_2_O (mean ± SD)	76.1 ± 39.9	77.9 ± 21.7	.879^b^
MEP pred (mean ± SD)	38.8 ± 15.5	39.3 ± 10.3	.914^b^
6MWD, m (mean ± SD)	362.2 ± 135.5	364.1 ± 86.7	.964^b^
6MWD pred, % (mean ± SD)	61.8 ± 19.5	64.0 ± 11.5	.711^b^
STST score (mean ± SD)	8.1 ± 3.5	10.0 ± 2.8	.113^b^
Fatigue (mean ± SD)	83.5 ± 25.1	93.9 ± 18.7	.209^b^

TG, training group; CG, control group; SD, standard deviation; M (IR), median (interquartile range); BMI, body mass index; F, female; ASA, American Society of Anesthesiologists; HT, hypertension; DM, diabetes mellitus; MELD, model for end-stage liver disease; Pred, predicted; MIP, maximal inspiratory pressure; MEP, maximal expiratory pressure; 6MWD, 6-minute walk distance; STST, sit-to-stand test.

The peripheral muscle strength values given in the table represent the dominant side.

^a^Chi-square; ^b^ *t*-tests; ^c^Mann–Whitney *U* test; ^†^Fisher’s exact test; *Significant differences between groups *P *< .05.

**Table 2.** The Comparison of the 4- and 8-Week Changes in the Outcome Measure Variables Between and Within Groups

**Variables**	**G**	**Preop**	**Fourth Week**	**Eighth Week**	*Preop* *—Fourth* *Week*		*Preop* *—Ēighth* * wk*		*Fourth to Eighth* *Week*	
*P*	*ES*	*P*	*ES*	*P*	*ES*
Shoulder flexion, kg	TG	11.2 ± 2.8	11.4 ± 2.5	13.5 ± 2.5	.720	0.06	.001*	0.59	.001*	0.59
	CG	11.6 ± 3.8	10.5 ± 4.0	10.3 ± 4.0	.102	0.30	.092	0.31	.589	0.10
*P*	.744	.445	.019*						
Shoulder abduction, kg	TG	11.0 (3.0)	11.3 ± 3.1	13.2 ± 2.9	.067	0.34	.001*	0.61	.002*	0.56
	CG	10.0 (6.0)	10.2 ± 4.3	10.0 ± 4.1	.283	0.20	.216	0.23	.726	0.06
*P*	.644	.417	.021*						
Knee extension, kg	TG	10.3 ± 2.2	11.8 ± 2.6	13.3 ± 2.5	.025	0.40	.001*	0.60	.001*	0.60
	CG	11.7 ± 3.1	12.1 ± 3.5	11.4 ± 3.1	.568	0.10	.751	0.06	.098	0.30
*P*	.168	.772	.080						
MIP, cmH_2_O	TG	64.0 ± 31.9	68.2 ± 20.9	76.4 ± 21.7	.460	0.14	.016*	0.44	.001*	0.62
	CG	69.4 ± 18.0	62.7 ± 18.8	66.3 ± 16.3	.078	0.32	.670	0.08	.078	0.32
*P*	.573	.458	.160						
MEP, cmH_2_O	TG	76.1 ± 39.9	62.0 (36.0)	89.0 (50.0)	.733	0.06	.047*	0.34	.001*	0.59
	CG	77.9 ± 21.7	66.0 (21.0)	69.0 (20.0)	.125	0.28	.336	0.17	.221	0.22
*P*	.879	.852	.059						
6MWD, m	TG	362.2 ± 135.5	422.5 ± 116.7	472.1 ± 98.1	.017*	0.43	.001*	0.59	.002*	0.57
	CG	364.1 ± 86.7	390.9 ± 116.4	407.1 ± 111.0	.244	0.21	.155	0.26	.233	0.22
*P*	.965	.463	.101						
STST	TG	8.1 ± 3.5	10.5 ± 2.7	14.0 (2.0)	.006*	0.50	.001*	0.62	.001*	0.58
	CG	10.0 ± 2.8	10.1 ± 3.4	10.0 (5.0)	.915	0.02	.048*	0.36	.018*	0.43
*P*	.114	.725	.082						
Fatigue	TG	83.5 ± 25.1	60.0 ± 19.9	41.0 (32.0)	.004*	0.52	.001*	0.62	.002*	0.57
	CG	93.9 ± 18.7	71.5 ± 27.3	81.0 (36.0)	.020*	0.43	.006*	0.50	.93	0.02
*P*	.209	.200	.009*						

Preop, preoperatively; ES, effect size; G, group; TG, training group; CG, control group; MIP, maximal inspiratory pressure; MEP, maximal expiratory pressure; 6MWD, 6-minute walk distance; STST, sit-to-stand test.

The peripheral muscle strength values given in the table represent the dominant side.

*Significant differences in changes between and within groups *P *< .05.

**Table 3.** Effect of Early Resistance Training on 6MWD and Muscle Strength Pretransplant, and 4 Weeks and 8 Weeks Posttransplant for Subjects in the TG (n = 15) and CG (n = 15).

**Weeks**	**Change Score**	**Difference in Change Score** **(95% CI)**	*P*	**MCID** **0.5 (Moderate) Substantial Meaningful Change**
**6MWD, m**				37.8
4-week change (0-4)		−33.5 (−106.0 to 38.9)	.350	
TG	60.3 ± 77.7			
CG	26.8 ± 111.9			
8-week change (0-8)		−66.8 (−132.8 to −0.8)	.047*	
TG	109.9 ± 75.6			
CG	43.1 ± 98.7			
**Knee extension, kg**				0.9
4-week change (0-4)		−1.1 (−2.9 to 0.8)	.240	
TG	1.5 ± 2.3			
CG	0.5 ± 2.6			
8-week change (0-8)		−3.3 (−5.0 to −1.5)	.001*	
TG	3.0 ± 1.8			
CG	−0.27 ± 2.7			
**Shoulder flexion, kg**				0.9
4-week change (0-4)		−1.3 (−3.1 to 0.5)	.136	
TG	0.2 ± 2.1			
CG	−1.13 ± 2.6			
8-week change (0-8)		−3.5 (−5.5 to −1.6)	.001*	
TG	2.3 ± 1.8			
CG	−1.3 ± 3.1			
**Shoulder abduction, kg**				0.9
4-week change (0-4)		−1.7 (−3.4 to 0.5)	.056	
TG	1.1 ± 2.1			
CG	−0.6 ± 2.4			
8-week change (0-8)		−3.7 (−5.5 to −2.0)	<.001*	
TG	2.9 ± 1.8			
CG	−0.8 ± 2.8			

TG, training group; CG, control group; 6MWD, 6-minute walk distance; MCID, minimal clinically important difference.

*Significant differences in change scores between groups *P *< .05.

## References

[1] Asrani SK Devarbhavi H Eaton J Kamath PS . Burden of liver diseases in the world. J Hepatol. 2019;70(1):151 171. 10.1016/j.jhep.2018.09.014) 30266282

[2] Turkish Ministry of Health, Transplantation, Dialysis and Monitoring Systems, Organ Decision Support Systems, Liver Transplants. Available at: https://organkds.saglik.gov.tr/dss/PUBLIC/Transplant_Liver.aspx; Accessed 29 October 2021.

[3] Diniz G Tugmen C Sert İ . Türkiye ve Dünyada Organ Transplantasyonu. “Türkiye ve dünyada organ transplantasyonu.” Tepecik Eğit ve Araşt Hast Dergisi. 2019;29(1):1 10. 10.5222/terh.2019.40412)

[4] Duarte-Rojo A Ruiz-Margáin A Montaño-Loza AJ Macías-Rodríguez RU Ferrando A Kim WR . Exercise and physical activity for patients with end-stage liver disease: improving functional status and sarcopenia while on the transplant waiting list. Liver Transpl. 2018;24(1):122 139. 10.1002/lt.24958) 29024353

[5] Boden I Skinner EH Browning L et al. Preoperative physiotherapy for the prevention of respiratory complications after upper abdominal surgery: pragmatic, double blinded, multicentre randomised controlled trial. BMJ. 2018;360:j5916. 10.1136/bmj.j5916) PMC5782401 29367198

[6] Pérez-Amate È Roqué i Figuls MR Fernández-González M Giné-Garriga M . Exercise interventions for adults after liver transplantation. Cochrane Database Syst Rev. 2018;11:CD013204. 10.1002/14651858.CD013204) PMC10201528 37204002

[7] Dhaliwal A Williams FR El-sherif O Armstrong MJ . Sarcopenia in liver transplantation: an update. Curr Hepatol Rep. 2020;19(2):128 137. 10.1007/s11901-020-00515-z)

[8] Janaudis-Ferreira T Mathur S Deliva R et al. Exercise for solid organ transplant candidates and recipients: a joint position statement of the Canadian Society of Transplantation and CAN-RESTORE. Transplantation. 2019;103(9):e220 e238. 10.1097/TP.0000000000002806) 31461743

[9] van den Berg-Emons RJ van Ginneken BT Nooijen CF et al. Fatigue after liver transplantation: effects of a rehabilitation program including exercise training and physical activity counseling. Phys Ther. 2014;94(6):857 865. 10.2522/ptj.20130402) 24557657

[10] Moya-Nájera D Moya-Herraiz Á Compte-Torrero L et al. Combined resistance and endurance training at a moderate-to-high intensity improves physical condition and quality of life in liver transplant patients. Liver Transpl. 2017;23(10):1273 1281. 10.1002/lt.24827) 28749550

[11] Schram A Ferreira V Minnella EM Awasthi R Carli F Scheede-Bergdahl C . In-hospital resistance training to encourage early mobilization for enhanced recovery programs after colorectal cancer surgery: a feasibility study. Eur J Surg Oncol. 2019;45(9):1592 1597. 10.1016/j.ejso.2019.04.015) 31053478

[12] Suetta C Magnusson SP Rosted A et al. Resistance training in the early postoperative phase reduces hospitalization and leads to muscle hypertrophy in elderly hip surgery patients—a controlled, randomized study. J Am Geriatr Soc. 2004;52(12):2016 2022. 10.1111/j.1532-5415.2004.52557.x) 15571536

[13] Krasnoff JB Vintro AQ Ascher NL et al. A randomized trial of exercise and dietary counseling after liver transplantation. Am J Transplant. 2006;6(8):1896 1905. 10.1111/j.1600-6143.2006.01391.x) 16889545

[14] VanWagner LB Uttal S Lapin B et al. Use of six-minute walk test to measure functional capacity After liver transplantation. Phys Ther. 2016;96(9):1456 1467. 10.2522/ptj.20150376) 27055540 PMC5009186

[15] Ashraf K Ayaz SB Yasmeen R Khan BU Fayyaz R Tassadaq N . Six-minute walk distance, ECOG Performance Status, and Modified Borg Scale scores in a cohort of Pakistani patients with noncancerous end-stage liver disease selected for liver transplant. J Pak Med Assoc. 2021;71(4):1162-1166. 10.47391/JPMA.05) 34125763

[16] Kim SG Lim DH Cho YHJ . Analysis of the reliability of the make test in young adults by using a hand-held dynamometer. J Phys Ther Sci. 2016;28(8):2238 2240. 10.1589/jpts.28.2238) 27630404 PMC5011568

[17] Rodrigues A Da Silva ML Berton DC et al. Maximal inspiratory pressure: does the choice of reference values actually matter? Chest. 2017;152(1):32 39. 10.1016/j.chest.2016.11.045) 27940276

[18] Tveter AT Dagfinrud H Moseng T Holm I . Health-related physical fitness measures: reference values and reference equations for use in clinical practice. Arch Phys Med Rehabil. 2014;95(7):1366 1373. 10.1016/j.apmr.2014.02.016) 24607837

[19] Worm-Smeitink M Gielissen M Bloot L et al. The assessment of fatigue: psychometric qualities and norms for the checklist individual strength. J Psychosom Res. 2017;98:40 46. 10.1016/j.jpsychores.2017.05.007) 28554371

[20] Senduran M Yurdalan SU Karadibak D Gunerli A . Haemodynamic effects of physiotherapy programme in intensive care unit after liver transplantation. Disabil Rehabil. 2010;32(17):1461 1466. 10.3109/09638280903531212) 20533874

[21] Al-Therapy Statistics, Comparing Two Sets of Data. Available at: https://www.ai-therapy.com/psychology-statistics/hypothesis-testing/two-samples?groups=0&amp;parametric=1; Accessed 29 July 2021.

[22] Norman GR Sloan JA Wyrwich KW . Interpretation of changes in health-related quality of life: the remarkable universality of half a standard deviation. Med Care. 2003;41(5):582 592. 10.1097/01.MLR.0000062554.74615.4C) 12719681

[23] Carey EJ Lai JC Sonnenday C et al. A North American expert opinion statement on sarcopenia in liver transplantation. Hepatology. 2019;70(5):1816 1829. 10.1002/hep.30828) 31220351 PMC6819202

[24] Philippe AG Borrani F Sanchez AM Py G Candau R . Modelling performance and skeletal muscle adaptations with exponential growth functions during resistance training. J Sports Sci. 2019;37(3):254 261. 10.1080/02640414.2018.1494909) 29972090

[25] Beyer N Aadahl M Strange B et al. Improved physical performance after orthotopic liver transplantation. Liver Transpl Surg. 1999;5(4):301 309. 10.1002/lt.500050406) 10388503

[26] Tomás MT Melo X Mateus É Gonçalves M Barroso E Santa-Clara H . A 5-year follow-up of the benefits of an exercise training program in liver recipients transplanted due to familial amyloidotic polyneuropathy. Prog Transplant. 2018;28(4):330 337. 10.1177/1526924818800033) 30261817

[27] Tomás MT Santa-Clara H Monteiro E Barroso E Sardinha LB . Effects of an exercise training program in physical condition after liver transplantation in familial amyloidotic polyneuropathy: a case report. Transplant Proc. 2011;43(1):257 258. 10.1016/j.transproceed.2010.12.025) 21335200

[28] Dunn MA Rogal SS Duarte-Rojo A Lai JC . Physical function, physical activity, and quality of life after liver transplantation. Liver Transpl. 2020;26(5):702 708. 10.1002/lt.25742) 32128971 PMC8063858

[29] Williams FR Berzigotti A Lord JM Lai JC Armstrong MJ . Review article: impact of exercise on physical frailty in patients with chronic liver disease. Aliment Pharmacol Ther. 2019;50(9):988 1000. 10.1111/apt.15491) 31502264

[30] Chascsa DM Lai JC Dunn MA et al. Patient and caregiver attitudes and practices of exercise in candidates listed for liver transplantation. Dig Dis Sci. 2018;63(12):3290 3296. 10.1007/s10620-018-5271-5) 30178285 PMC6532051

